# The Impact of Selenium Deficiency on Cardiovascular Function

**DOI:** 10.3390/ijms221910713

**Published:** 2021-10-02

**Authors:** Briana K. Shimada, Naghum Alfulaij, Lucia A. Seale

**Affiliations:** Pacific Biosciences Research Center, University of Hawaii at Manoa, Honolulu, HI 96822, USA; bkshimad@hawaii.edu (B.K.S.); alfulaij@hawaii.edu (N.A.)

**Keywords:** selenium, cardiovascular, heart, selenoproteins, Keshan’s Disease

## Abstract

Selenium (Se) is an essential trace element that is necessary for various metabolic processes, including protection against oxidative stress, and proper cardiovascular function. The role of Se in cardiovascular health is generally agreed upon to be essential yet not much has been defined in terms of specific functions. Se deficiency was first associated with Keshan’s Disease, an endemic disease characterized by cardiomyopathy and heart failure. Since then, Se deficiency has been associated with multiple cardiovascular diseases, including myocardial infarction, heart failure, coronary heart disease, and atherosclerosis. Se, through its incorporation into selenoproteins, is vital to maintain optimal cardiovascular health, as selenoproteins are involved in numerous crucial processes, including oxidative stress, redox regulation, thyroid hormone metabolism, and calcium flux, and inadequate Se may disrupt these processes. The present review aims to highlight the importance of Se in cardiovascular health, provide updated information on specific selenoproteins that are prominent for proper cardiovascular function, including how these proteins interact with microRNAs, and discuss the possibility of Se as a potential complemental therapy for prevention or treatment of cardiovascular disease.

## 1. Introduction

Selenium (Se) is an essential trace element necessary for a variety of biological functions in animals, including cardiovascular function. Se deficiency has been linked to different cardiovascular diseases, including cardiomyopathies such as Keshan’s disease (KD) [1,2], heart failure [3,4], and myocardial infarction [5]. Although there has been increasing evidence of the importance of Se for optimal cardiovascular function, the role of Se in cardiovascular syndromes, particularly under dietary Se deficiency, remains only partially understood.

The main effects of Se are manifested when it is part of the catalytic center of selenoproteins in the form of the amino acid selenocysteine. Se compounds obtained in the diet are rapidly metabolized via the trans-selenation pathway or reduced in the presence of glutathione (GSH), to allow for the production of the common intermediate, selenocysteine (Sec) [6], an amino acid that is then incorporated into peptide chains to form selenoproteins. Interestingly, for Sec to be used in selenoprotein translation, it needs to be decomposed into selenide by an enzyme called selenocysteine lyase (SCLY) [7], which will then allow for its synthesis attached to a specific tRNA, a process thoroughly reviewed elsewhere [8]. Briefly, the selenophosphate synthetase enzyme (SEPHS2) utilizes the selenide released from SCLY to produce monoselenophosphate for Sec biosynthesis. Sec is then attached to its tRNA and utilized in selenoprotein translation. 

Overall, 25 genes encoding for selenoproteins have been found in humans, and 24 in rodents [9]. Selenoproteins are expressed in a wide range of tissues and have a variety of functions, including curbing free radicals, controlling calcium flux, and maintaining thyroid hormone levels [10]. Several of these selenoproteins have vital roles in the heart, including glutathione peroxidases (GPXs) [11,12], iodothyronine deiodinases (DIOs) [13], thioredoxin reductases (TXNRDs) [14], selenoprotein W (SELENOW) [15], selenoprotein P (SELENOP) [16], MsrB1 (previously known as selenoprotein R) [17], selenoprotein T (SELENOT) [18], and selenoprotein K (SELENOK) [19]. Low Se levels disrupt the synthesis of a subgroup of stress-induced selenoproteins, including GPX1, leading to the shortage of one or more of these crucial proteins in the heart [3], with a potential impact on overall cardiovascular health [20]. In fact, removal of selenoproteins by deleting Sec tRNA in the heart and skeletal muscle resulted in sudden cardiac arrest due to increased oxidative stress and inflammation [21]. Nevertheless, Se metabolism and the molecular mechanism by which selenoproteins participate in and regulate heart function has not been completely determined, particularly under Se deficiency. Therefore, a better understanding of the molecular mechanisms behind Se and selenoproteins involvement in cardiac function is needed to explain how Se deficiency may contribute to the development of cardiovascular disease. As selenoproteins involvement in the heart have been reviewed previously [3,10,22], we aim in this review to briefly cover select key selenoproteins and focus mainly on recent studies (within the past five years), particularly the current knowledge about Se metabolism and cardiac selenoproteins in the context of cardiovascular diseases, updating what has been previously discussed in the literature [10,22]. We focus on the role of Se metabolism and some selenoproteins in the heart function, its involvement in the pathogenesis of cardiovascular diseases, and finally debate Se as a potential therapeutic or preventative supplement for cardiovascular syndromes.

## 2. Methods

The following criteria were used to select references for this review: 

Databases searched: Pubmed (NLM).

Keywords searched: selenium, selenium metabolism, selenium deficiency, selenoproteins, cardiovascular diseases, myocardial infarction, ischemia reperfusion injury, atherosclerosis, oxidative stress, Keshan’s Disease, heart, cardiovascular, glutathione peroxidase, thioredoxin reductase, iodothyronine deiodinases, microRNAs, Selenoprotein P, Selenoprotein T, Selenoprotein K, Selenoprotein W, selenite, selenomethionine, Selenoprotein MsrB1, Selenoprotein S, and Selenoprotein P.

## 3. The relationship between Se Deficiency and Cardiovascular Health

The first evidence of Se involvement in cardiovascular function came from the discovery that Se deficiency was involved in KD, a severe form of cardiomyopathy that is sometimes fatal. It is an endemic disease, first found in Keshan County in northeast China. KD is characterized by myocardial necrosis, calcification, and fibrosis, leading to various clinical manifestations such as cardiogenic shock, cardiac arrhythmias, and congestive heart failure [23]. Observations that KD shared similar morphology to white muscle disease, a degenerative cardiac and skeletal muscle disease found in foals from Se-poor areas [24], provided a clue that Se deficiency may play a role in KD [2]. Supporting this hypothesis, administration of oral Se reversed Se deficiency and improved outcomes of KD [25,26]. Although there is some debate about whether Se deficiency is the primary cause of KD as there are several additional underlying causes, it remains until now the most convincing possibility [1,26].

Further strengthening the argument that Se is important in cardiovascular function is that Se deficiency also contributes to the pathogenesis of other cardiomyopathies and heart failure. A recently reported rare case of dilated cardiomyopathy in a young boy was attributed to chronic starvation and a severe Se deficiency [27]. Notably, nutritional support with Se supplementation resulted in the reversal of the disease. Another case recently reported involved a malnourished woman with Se deficiency that resulted in cardiomyopathy, with the condition being reversed by Se treatment [28]. 

Se deficiency also is associated with heart failure [4,29,30,31]. Serum Se concentrations measured in a large European cohort of patients with worsening heart failure were low in 70% of these patients. Low Se levels in these patients were associated with a poorer quality of life, impaired exercise capacity, and an inferior prognosis with worsening heart failure [4]. Moreover, this same study demonstrated human cardiomyocytes generated from human pluripotent stem cells (hPSCs) cultured in low Se resulted in reduced mitochondrial function and increased reactive oxygen species (ROS), thereby showing impairment of key metabolic processes. Despite the increasing evidence of Se involvement in the pathogenesis of cardiovascular diseases, molecular mechanisms linking Se deficiency to cardiovascular diseases remain to be elucidated.

### Potential Mechanisms behind Se Deficiency and Cardiovascular Disease

With the likely linkage between Se and cardiovascular health, studies have been conducted to examine the mechanism behind this association. Se has been suggested to participate in cell survival [32,33,34], a role reviewed recently [35]. The key findings in the studies connecting Se to cardiomyocyte survival is that, predominantly, Se deficiency increased apoptosis while inhibiting autophagy in the heart. Pro-apoptotic proteins, such as cleaved caspases-3, -8, and -9 and Bax, were upregulated, while anti-apoptotic ones, such as BCL-2, were reduced [32,33,34]. Combined, these findings suggest that Se regulates cardiomyocyte apoptosis. 

Additional reports suggest a role of microRNAs (miRNAs) during Se deficiency in sustaining cardiovascular function. MiRNAs are an emerging topic in cardiovascular research as they have been shown to play an important role in a variety of cardiovascular diseases. These roles include cell survival, inflammation, and curbing of oxidative stress. Therefore, it has been hypothesized that miRNAs could also be regulating or contributing in cardiomyopathies related to Se deficiency. Studies using miRNA array or miRNA specific omics identified potential miRNAs that could be involved in such regulation. One study examined miRNA expression in Se-deficient rats using an miRNA array and found the cardiac dysfunction of the Se-deficient rat to be associated with five upregulated microRNAs, namely, miR-374, miR-16, miR-199a-5p, miR-195, and miR-30e, as well as three downregulated miRNAs, namely, miR-3571, miR-675, and miR-450a [36]. Another report used microRNAome analysis to examine miRNAs in the myocardium of Se-deficient chickens and found miR-2954 to have increased expression [37]. Bioinformatic analysis predicted phosphoinositide 3-kinase (PI3K), a key protein regulating cell apoptosis and autophagy, as the target gene of miR-2954. Overexpression of this miR led to autophagy and apoptosis of myocardial cells through the regulation of the PI3K pathway. This same microRNAome analysis also identified miR-200a-5p, and its target gene for ring finger protein 11 (RNF11), as triggering necroptosis in cardiomyocytes [38]. RNF11 is involved in the necroptosis pathway, a form of programmed necrosis. Knockdown of miR-200a-5p in Se-deficient cardiomyocytes resulted in enhanced cell survival after treatment with z-VAD-fmk, a necroptosis inducer. It is interesting to note that the miRNAs found by these microRNAomics are mainly involved in cell death pathways. This is consistent with the previous findings that Se deficiency leads to the increase in pro-cell death pathways. Further investigation into the interplay between miRNAs and Se in the heart is needed to determine how both contribute to molecular mechanisms leading to cardiomyopathy and other cardiovascular diseases.

## 4. Se Metabolism in the Heart

**Figure 1:**
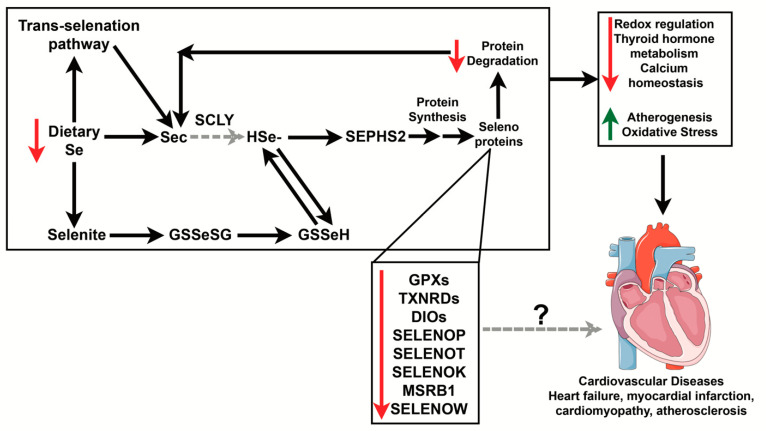
Potential mechanism of how Se deficiency impacts Se metabolism and contributes to cardiovascular diseases. Se acquired through the diet can go through the trans-selenation pathway and be directly converted to Sec, becoming hydrogen selenide. In cases of low Se, stress-responsive selenoprotein synthesis may be affected. Those selenoproteins with known function in the heart are shown above but it is still unknown how some of these proteins contribute to the development of cardiovascular disease in cases of Se deficiency. Decreased levels of Se may negatively impact redox regulation, thyroid hormone metabolism, and calcium flux while increasing atherogenesis and oxidative stress. This, in turn, may lead to several cardiovascular diseases, including heart failure, myocardial infarction, cardiomyopathy, and atherosclerosis. Red arrows indicate processes and proteins that are decreased while green arrows indicate processes that are increased during Se deficiency. Black arrows point to known relationships, and dashed gray lines indicate the relationships that have not been established yet in the heart. The “?” indicates mechanisms that have not been determined. Se, selenium; Sec, selenocysteine; HSe–, hydrogen selenide; SEPHS2, selenophosphate synthetase 2; GPX, glutathione peroxidase; TXNRDs, thioredoxin reductases; DIO, iodothyronine deiodinases; SELENOP, selenoprotein P; SELENOT, selenoprotein T; SELENOK, selenoprotein K; MSRB1, methionine sulfoxide reductase B1; SELENOW, selenoprotein W. Heart was used from Servier Medical Art (smart.servier.com).

Se is an essential trace element that supports heart function [39]. It is vital to maintain adequate Se levels in the body as both low and excess levels of Se can have detrimental effects to cardiovascular health. As we previously mentioned, Se deficiency has been linked to cardiomyopathies, including KD and heart failure [3,29,40,41]. Excess Se intake, on the other hand, may result in severe toxicity and cardiac symptoms that may be fatal [42,43]. Although it is clear that adequate Se levels maintain normal cardiovascular function, a comprehensive understanding of the molecular mechanisms linking how Se metabolism contributes to cardiovascular disease is still lacking. Se exists in both inorganic and organic chemical forms, entering the food chain through plants and microorganisms via uptake by sulfate transporters. These forms include selenomethionine (SeMet), Sec, selenite, selenate, and selenious acid, among others. Depending on the chemical form of Se ingested, the Se compound may need to be either reduced (inorganic forms) or metabolized using the trans-selenation pathway (most organic forms) before it can be incorporated into a selenoprotein as Sec [7]. These mechanisms of Se metabolism have been demonstrated to occur in cells that are highly dependent on Se, such as hepatocytes [44,45,46]; however, they have not been determined in cardiomyocytes. Below we summarize how Se might be metabolized in the heart and how a diet deficient in Se may contribute to the development of several cardiovascular diseases (Figure 1). This will be discussed in greater detail in subsequent sections.

### 4.1. Trans-Selenation Enzymes in the Heart

An essential component of Se metabolism is the trans-selenation pathway that produces Sec as a byproduct of selenomethionine (SeMet) metabolism [44]. There are three main enzymes involved in this process: cystathionine beta-synthase (CBS), cystathionine gamma-lyase (CGL), and SCLY. CGL and CBS sequentially convert selenohomocysteine to Sec while SCLY decomposes L-Sec into L-alanine and hydrogen selenide (H_2_Se) in various mammalian tissues [7]. It remains unknown whether SCLY has a similar function in the heart; however, CGL and CBS have both been purported to protect the heart against ischemic damage [47,48]. We will discuss the function of these enzymes and their potential role in the heart below. 

#### 4.1.1. Cystathionine Beta-Synthase (CBS)

CBS is the first enzyme in the transsulfuration pathway that catalyzes the conversion of serine and homocysteine to cystathionine and H_2_O [49]. Functional knowledge about this enzyme’s action in the heart is poor; however, a recent study, using a combination of sodium thiosulfate and a CGL inhibitor, propargylglycine (PAG), to treat the rat cardiac myoblast cell line H9C2 cells undergoing hypoxia and reoxygenation, or rat hearts undergoing ischemia/reperfusion (I/R), revealed a partial recovery from these states [48], indicating that CBS may play a protective role as the function of CGL was inhibited by PAG, therefore being ruled out as a player in this protective mechanism in the heart. 

#### 4.1.2. Cystathionine Gamma-Lyase (CGL)

CGL is a sulfide (H_2_S)-producing enzyme with L-cysteine as its main substrate, and therefore a member of the transsulfuration pathway that follows the methionine cycle. In the heart, specific overexpression of CGL protected against heart failure induced by permanent coronary ligation and significantly improved survival in mice [47]. Exogenous H_2_S administration at the time of reperfusion in mice also protected against detrimental left ventricular remodeling that can lead to heart failure, and preserved cardiac function by attenuating oxidative stress and mitochondrial dysfunction [47]. H_2_S administration also led to isoproterenol–caffeine-induced left ventricular hypertrophy in rats through upregulation of myocardial CGL [50]. 

#### 4.1.3. Selenocysteine Lyase (SCLY)

SCLY was first identified as an enzyme that decomposes L-Sec into L-alanine and H_2_Se in eukaryotes [51]. Sec for SCLY decomposition is typically acquired in the diet, produced via the trans-selenation pathway or released from selenoprotein degradation [52]. This enzyme was first purified from pig liver [51], and has been detected in several human tissues, including the heart; however, it is found at modest levels [53], and has a relatively unexplored role in the heart. Interestingly, one study revealed that after treating H9C2 cells with the H_2_Se homologue, hydrogen sulfide (H_2_S), oxidative stress was significantly attenuated [54]. Moreover, H_2_S treatment increased SCLY/H_2_Se signaling and resulted in the increased activity and expression of multiple selenoproteins, including GPX1 and TXNRD2. Therefore, it is likely that SCLY plays a role in the heart, but whether it responds also to Se deficiency, as it does in other tissues, and whether it is involved in the pathogenesis of cardiovascular diseases, remains to be explored. 

## 5. Selenoproteins in the Heart

Selenoprotein involvement in heart function has been well documented and reviewed previously [3,10,22]. Therefore, as abovementioned, we will briefly cover select key selenoproteins and focus mainly on recent studies (within the past five years). These proteins are discussed below:

### 5.1. Glutathione Peroxidases (GPXs)

GPXs neutralize reactive oxygen and nitrogen species by catalyzing the reduction of hydrogen peroxides (H_2_O_2_) to water. There are five Sec-containing GPX enzymes in humans: GPX1, GPX2, GPX3, GPX4, and GPX6 [55]. Of the five, GPX1 is the most well-documented in cardiac tissues and known to be cardioprotective against I/R injury in mice [11]. Mice lacking GPX1 exhibited increased susceptibility to I/R injury due to increased apoptosis. GPX1 also regulates oxidative stress; hypoxia inducible factor (HIF)-2a knockout (KO) mice exhibited increased oxidative stress that was associated with low levels of GPX1 [56]. Interestingly, GPX1 is a stress-responsive selenoprotein, i.e., it is tightly regulated by Se levels. It is possible that Se deficiency may contribute to downregulate its expression, deteriorating the heart capacity to respond to oxidative stress, contributing to poor cell survival. 

GPX3 and GPX4 are also highly expressed in the heart although their role in cardiac tissue is not as clear as GPX1. GPX3, a mostly plasma GPX enzyme, scavenges ROS in extracellular spaces and in the vasculature. It has been shown to protect against stroke by regulating the bioavailability of nitric oxide [57] and was also found to be upregulated during myocardial hypertrophy [17]. GPX4 protects cellular lipids from oxidative damage. Although GPX4 has a crucial role in the cellular antioxidant defense, it is still unclear what the specific mechanism in which GPX4 participates is being affected in times of cardiac stress. In other organs, GPX4 is resistant to Se deficiency, although it is still unknown if this is true in the heart. GPX4 overexpression in the mitochondria protected neonatal rat cardiomyocytes against ischemic damage [58]. It has also been shown that GPX4 protects cellular lipids from oxidative damage during hypertrophy [17]. More recently, a proteomic study revealed that the downregulation of GPX4 exacerbates ferroptosis, a form of iron-dependent nonapoptotic cell death, during acute myocardial infarction (MI) [59]. This finding is supported by other studies that have revealed the ferroptosis inducer erastin to downregulate GPX4 expression in H9C2 rat cardiac myoblasts [60]. Moreover, inhibition of ferroptosis using another ferroptosis inhibitor, liproxstatin-1, protected hearts against ischemic damage and restored GPX4 expression [61]. These interesting studies suggest that increasing GPX4 expression in the heart may help prevent ferroptosis, a contributor to myocardial infarction (MI). Inhibition of ferroptosis has been repeatedly demonstrated to protect the heart against ischemic damage and may suggest a new role for GPX4 as regulating cardiovascular function [62].

### 5.2. Iodothyronine Deiodinases (DIOs)

The main function of the DIOs is to regulate thyroid hormone levels [63]. There are three DIO isoforms: DIO1, DIO2, and DIO3. DIO1 and DIO2 catalyze the release of outer ring iodine from thyronine hormones to convert the prohormone thyroxine (T4) to the active 3-3’-5-triiodothyronine (T3) form, while DIO3 inactivates both T3 and T4 by removing an inner ring iodine from the molecule. DIO2 is highly expressed in the heart as well as brown adipose tissue and the pituitary. DIO1 is predominantly expressed in the liver, kidneys, and thyroid, as well as many other tissues, while DIO3 is expressed in the placenta, uterus, brain, and fetal tissues [64]. Dysregulation of thyroid hormones mainly impacts myocardial development and differentiation, although there are indications that these hormones may also be involved in cardiac hypertrophy and I/R injury [65,66]. Overexpression of DIO2 in mice enhanced myocardial contractility in a pressure-overload hypertrophy model that was accompanied by increased expression of SERCA2a and improved contractility, likely due to increased sarcoplasmic reticulum (SR) Ca^2+^ uptake [65]. As thyroid hormones are known to regulate SERCA expression in skeletal muscle [67] and heart [68], and deiodinases control thyroid hormones, it is not surprising that upregulation of DIO2 also impacts SERCA expression.

Thyroid hormones also attenuate cardiac remodeling after MI [66,69]. Reduced plasma levels of these hormones, particularly T3, is associated with ventricular failure and increased expression of DIO3 [70,71]. Consistent with these earlier findings, a recent report used an infusion of 6 μg/kg/day of T3 prior to subjecting rats to I/R injury, and the T3 treatment improved cardiac function following the injury [13]. Interestingly, DIO1 and DIO2 expression were significantly increased in the area at risk (the area around the ischemic injury) in rats given T3 before I/R. In the remote zone (the region distant to the ischemic injury), DIO1 and DIO3 were downregulated in sham and rats not given T3 prior to I/R [13]. This suggests novel roles for all three isoforms of the DIOs in I/R injury. Additionally, an interesting study that examined the regulation of an miRNAs on DIO3 revealed that miR-214 was found to be co-expressed with DIO3 in cardiomyocytes post-MI. Fascinatingly, DIO3 expression preceded miR-214 expression in the left ventricle and locally suppressed the known cardioprotective effect of hormone T3. High expression of T3 significantly reduced miR-214, suggesting a possible negative feedback loop where miR-214 controls DIO3 expression [72]. This is a potential novel area for the field to expand upon, understanding how various miRNAs may fine-tune DIO expression and, consequently, T3 availability, in the context of cardiovascular diseases such as myocardial infarction. 

### 5.3. Thioredoxin Reductases (TXNRDs)

In mammals, TXNRDs mainly regulate intracellular redox reactions for functions as varied as DNA synthesis, redox status of transcription factors (e.g., NF-kB and AP-1), immunomodulation, and regulation of apoptosis [73]. Thioredoxins are utilized by TXNRD enzymes that use NADPH/H+ as a reducing agent to regenerate reduced thioredoxins, which are used to reduce oxidized cysteine residues in cellular proteins. There are three TXNRD enzymes, TXNRD1, TXNRD2, and TXNRD3, and all have well-documented roles in cardiovascular function, such as mitigating oxidative stress in response to pressure overload-induced hypertrophy [14] and ameliorating left ventricular remodeling [74]. 

Recent studies have continued to examine the impact of the thioredoxins (Trxs) and TXNRDs in multiple cardiovascular diseases and during oxidative stress in rodent and cell models with one study exploring what happens to selenoprotein expression after knockdown of Trx in a Se-deficient chicken cardiomyocyte model [75]. Chicken cardiomyocytes were given low Se (0.033 mg/kg) and treated with siRNA to knockdown thioredoxins. By qRT-PCR, mRNA expression of several selenoproteins, including all three TXNRDs, were significantly reduced [75], indicating that the thioredoxin levels may be important in cardiomyocytes to regulate TXNRDs. However, no functional analysis was performed in this study; therefore, it is unknown whether this was a maladaptive or adaptative response. In another study, BALB/C mice were provided with excess iron to determine how iron affects the thioredoxin system in the mouse heart [76]. It was discovered that iron overload altered protein expression of the thioredoxin-interacting protein (TXNIP) and TXNRD1 but that gene expression of these proteins remained unchanged. It would be interesting if the field focused on how TXNRDs and Trxs interact when there is inadequate Se in the heart as there are few studies focusing on how Se deficiency impacts the function of TXNRDs and Trxs. 

### 5.4. Selenoprotein P (SELENOP)

SELENOP is of particular interest as Se is largely bound to circulating SELENOP in the plasma, providing Se to several tissues. Nevertheless, there are conflicting reports in the literature as to whether SELENOP may be beneficial or detrimental to the heart. Low SELENOP levels are associated with increased risk of mortality in acute heart failure patients and all-cause cardiovascular mortality [77,78]. A decrease in circulating levels of SELENOP was also associated with a greater risk of metabolic syndrome in patients with documented cardiovascular disease [79]. Despite this clinical association, it is unclear if SELENOP is indeed necessary to prevent cardiovascular disease, as SELENOP KO mice subjected to I/R injury exhibited significantly reduced infarct sizes, indicating that less tissue was damaged, and cardiac apoptosis compared to WT mice. This reduction in tissue injury corresponded to an increase in phosphorylation of several proteins involved in the reperfusion injury salvage kinase (RISK) pathway, such as Akt and Erk [16]. SELENOP levels were also higher in patients with cardiogenic shock and complicating acute MI, and thirty-day mortality was significantly higher in patients with SELENOP levels above the 75th percentile 3 days post-MI [80].

### 5.5. Selenoprotein T (SELENOT)

SELENOT has been shown to be involved in cardiac development, as it has showed a dramatically increased expression following an ex vivo Langendorff I/R model. This upregulation suggests that SELENOT may not be required when heart function is normal, but can be activated in times of stress [18]. It was suggested that SELENOT regulates calcium homeostasis, and this upregulation effect may be a compensation by the heart to protect against calcium overload-mediated cell death [81]. It should be noted that studies analyzing SELENOT are very limited, and thus any current conclusions regarding the role of SELENOT in I/R injury should be taken with caution.

### 5.6. Selenoprotein K (SELENOK)

In the heart, SELENOK was first identified to have an antioxidant effect in cardiomyocytes [19]. Overexpression of SELENOK in cardiomyocytes using a recombinant adenovirus system attenuated ROS and protected against oxidative stress induced by H_2_O_2_ [19]. In addition to this protein’s antioxidant capabilities, SELENOK has also been identified as a regulator of calcium flux in immune cells [82]. In the heart, immune cells may exacerbate tissue damage after MI due to increased inflammation and these cells contribute to the development of atherosclerosis. Interestingly, SELENOK itself may also play a role in atherogenesis. SELENOK expression was detected in aortic plaques, particularly in macrophages, and SELENOK KO animals exhibited reduced atherosclerosis as indicated by lesion formation, which may contribute to foam cell formation and atherogenesis [83].

### 5.7. Selenoprotein MsrB1 

The role of selenoprotein MsrB1 in cardiac health has been reviewed previously [10]; therefore, we will only provide an update here. MsrB1 reduces methionine sulfoxide, a byproduct of ROS oxidation of methionine residues. MsrB1 was identified as a selenoprotein highly expressed in the heart during cardiac stress after T3 and isoproterenol treatment [17]. It is thought that MsrB1 may be upregulated as a compensatory response to prevent oxidative damage or induced to regulate actin remodeling during myocardial hypertrophy. Additional studies are required to assess the molecular mechanism behind MsrB1 upregulation during hypertrophy. 

### 5.8. Selenoprotein W (SELENOW) 

Like other selenoproteins, SELENOW serves as an antioxidant [84], acting in calcium regulation and redox regulation [85]. It may also be involved in muscle growth and differentiation as this protein was shown to be highly expressed in proliferating myoblasts but not differentiated ones [86]. SELENOW levels are highest in the heart, muscle, and brain in sheep and primates, but is low in rodent hearts [87], suggesting that its role in the heart is species-specific, potentially only in non-rodent mammals. There are few studies to confirm this, however, SELENOW expression was increased by Se treatment in myocardial chicken cells [15], and is therefore a cardiac selenoprotein sensitive to Se levels. Future studies can determine if Se deficiency modulates SELENOW actions and what role this enzyme might play in heart function. 

### 5.9. Selenoprotein S (SELENOS)

Another selenoprotein that may be involved in cardiovascular function is SELENOS. In Finnish and Chinese cohorts, single nucleotide polymorphisms (SNPs) were associated with increased risk for coronary heart disease and ischemic stroke [88], as well as type 2 diabetes, which carries an increased risk of cardiovascular disease [89]. Nevertheless, despite the association between certain SNPs and cardiovascular disease, there have not been follow-up studies and not much is known about the role of this protein in the heart. 

## 6. Se Supplementation for Mitigation of Cardiovascular Diseases

As mentioned before, Se plays a role in the pathogenesis and responses of several cardiovascular diseases, including cardiomyopathy, myocardial infarction, cardiovascular stress response, and hypertrophy, particularly under deficient intake. From early on, Se deficiency has been implicated in KD and heart failure, and over time it was shown that a subset of selenoproteins play vital roles during I/R injury, cardiomyopathy, and heart failure. Excess Se has been connected to decreased cardiac output in pigs [90] and increased incidence of type 2 diabetes [91], a syndrome that greatly increases the risk of cardiovascular disease [92]. Below, we will explore recent studies that investigate the effects of Se supplementation in the context of several cardiovascular diseases.

Clinically, low Se levels are associated with higher rates of all-cause mortality due to heart failure [4], increased rates of myocardial infarction [5], and cardiomyopathies [27,93]. It is therefore unsurprising that several studies have focused on Se supplementation to mitigate cardiovascular disease despite mixed results, likely due to a host of factors, including the different pharmacokinetics of various forms of Se, the range and duration of treatment, and whether Se was used alone or in combination with another therapy. Among the cardiovascular conditions that have employed Se supplementation in an attempt to mitigate the disease pathogenesis in animal/cell models or clinically, include all-cause cardiovascular mortality [94], peripartum cardiomyopathy [95], I/R injury [96,97], atherosclerosis [98], and coronary heart disease [99]. More rigorous studies are needed to determine solely the effects of Se supplementation on specific cardiovascular mechanisms and prognostic outcomes. Moreover, studies comparing the pharmacokinetics of multiple chemical forms of Se supplementation in heart health are lacking. We will discuss the studies so far that have utilized Se as a supplement to mitigate various cardiovascular diseases and discuss why their results have varied significantly.

### 6.1. Se Supplementation in Myocardial Infarction and I/R Injury

Se plays vital roles during MI, such as the reduction of ischemic injury and left ventricular remodeling, likely due to decreased oxidative stress. It was recently reported that Se deficiency was found in the majority of MI patients [5,100]. Moreover, patients with high Se levels exhibited the lowest prevalence of cardiovascular outcomes, including MI in an Inuit cohort in Canada [101]. This suggests that strategies aiming at Se adequacy, such as Se supplementation in deficient individuals or populations, may mitigate ischemic damage to the heart. Nevertheless, there have been limited mechanistic studies addressing this possibility. Pretreatment of rats with Se prior to in vivo I/R injury led to better cardiovascular outcomes [96]. Another study found that radioactive Se 75 in the form of selenide targets damaged tissue after myocardial I/R injury [97]. Based on markers of cell damage, such as neutrophil accumulation, cardiac troponin levels, and measurements of cardiac function, it was concluded that selenide treatment in solution reduced damage to the heart [97]. Intriguingly, this was the only inorganic form of Se that was capable of doing so as treatment with reduced Se, oxidized Se, and selenite had no effect. As different forms of Se have varying pharmacokinetics, besides also being absorbed differentially by the gut microbiome [102], these differences serve as reminders when investigating whether a particular form of Se may be suitable to use as a supplement to protect the heart from ischemic damage. 

The different pharmacokinetics of Se compounds may be one reason why Se supplementation as a strategy to prevent or mitigate I/R injury has had mixed results, although studies in this area are limited. A recent report demonstrated SeMet was effective at preventing necrotic cell death in a rat H9C2 myoblasts [103]. However, SeMet only modestly improved cardiac function and was incapable of preventing remodeling following I/R injury in Wistar rats. Another chemical form of Se, the inorganic selenite, was used in rats undergoing I/R injury during cardiovascular surgery. Selenite suppressed tissue damage as indicated by markers of cardiac injury such as lactate dehydrogenase and cardiac troponin but did not prevent the production of inflammatory cytokines such as IL-6 and TNF-α [104]. There have been combination therapies as well, although the added complication of mixing compounds compromises the ability to distinguish if, and which, Se form is indeed the primary effector for improving I/R injury outcomes. One of these tested therapies utilized aloe vera biomacromolecules conjugated with Se, finding that pretreatment of the compound decreased infarct sizes, cardiac injury markers, and cardiomyocyte apoptosis [105]. Clinically, a combination therapy of Se and coenzyme Q10 was purported to have beneficial effects in preventing all-cause mortality from cardiovascular disease, including in those individuals with ischemic heart disease. Moreover, this effect continued for 12 years after supplementation was stopped [94,106]. While intriguing, this study is problematic in that the protection from Se is unclear, since the subjects were also treated with coenzyme Q10. Therefore, studies that focus solely on the effect of Se as a therapeutic treatment to determine if Se supplementation is cardioprotective in MI and I/R injury are needed.

### 6.2. Se Supplementation in Atherosclerosis

Atherosclerosis is a disease of the arteries that develops slowly over many years and is characterized by fat and cholesterol deposition in large and medium sized arteries. Mechanistically, atherosclerosis develops through the increased transcytosis of low-density lipoprotein (LDL), increased inflammation, endothelial dysfunction, and leukocyte migration into the arterial intima where mononuclear phagocytes proliferate and engulf lipids. After enough lipids have been engulfed, these phagocytes eventually transform into foam cells. Excessive foam cell formation results in the accumulation of cholesterol esters, and is termed a fatty streak. In the fatty streak, lymphocytes and macrophages secrete inflammatory cytokines and enhance vascular smooth muscle cell (VSMC) migration into the intima. This, in turn, thickens the arterial wall, and the fatty streak evolves into a stable plaque. Eventually, in more advanced stages, these VSMCs proliferate, secrete extracellular matrix proteins, and generate a fibrous cap, while the apoptotic and necrotic cell death of foam cells produces what is termed a necrotic core [107]. Increased oxidative stress is considered a major contributor to the development of atherosclerosis; ROS are key mediators of inflammatory signaling pathways that lead to atherogenesis and the formation of the fatty streak [108]. As selenoproteins are heavily involved in the protection against oxidative stress, it is likely that Se plays a role in atherogenesis. Indeed, several studies have previously shown in various animal models that Se supplementation prevents atherosclerosis [109,110,111,112]. More recently, SeMet supplementation was tested in a mouse model of atherosclerosis [98]. Apolipoprotein E-deficient (ApoE^−/−^) mice were placed on a high-fat diet without supplemental Se or were given a high fat diet with 2 mg/kg of SeMet for 6 or 12 weeks. After either 6 or 12 weeks of SeMet treatment, the study demonstrated a significant decrease in lesion burden, which was accompanied by the formation of a more stabilized plaque. To concur with a role of selenoproteins in this protective effect, GPX1 expression and activity were increased. Interestingly, an insertion and deletion (INDEL) SNP in SCLY, the enzyme that catalyzes a key step in SeMet metabolism, has also been linked with increased risk to develop atherosclerosis in Mexican-American subjects [113].

More recently, studies have focused on using engineered forms of Se such as Se nanoparticles [114,115] and Se quantum dots [116] to determine if they prevent atherosclerosis in murine and rat models of atherosclerosis. Animals treated with 50 mg/kg/day of Se nanoparticles (SeNPs) for 8 weeks significantly attenuated vascular injury [114]. This was associated with significantly lower levels of triglycerides, total cholesterol, and LDL-cholesterol in the serum of mice given the SeNPs. Oxidative stress, as measured by serum malondialdehyde (MDA) levels, was significantly lower compared with the control, and corresponded with an increase in the activity of antioxidant enzymes, GPX, and super oxide dismutase (SOD), suggesting SeNPs may mitigate the development of atherosclerosis, at least in a murine model. Indeed, the other study using SeNPs by the same group demonstrated that SeNP administration for 12 weeks significantly decreased atherosclerotic lesions in ApoE^−/−^mice [115]. This was again associated with a decrease in hyperlipidemia and oxidative stress. Finally, rats or ApoE^−/−^ mice on a high-fat diet were treated with Se quantum dots (SeQDs) at a dose of 0.1 mg/kg/day, or a combination of lithium chloride (LiCl) and quantum dots [116]. SeQDs protected against endothelial dysfunction in rats, an effect associated with the inhibition of NHE1 by LiCl, a pathway known to protect against endothelial dysfunction. In ApoE^−/−^ mice, SeQDs decreased the serum nitric oxide levels, attenuated endothelial dysfunction, and inhibited the formation of atherosclerotic plaques. It is evident that, at least in rodent models, Se supplementation is beneficial to protect against atherosclerosis; however, it remains to be seen if this is also true in humans, as clinical studies are currently limited. Moreover, the mechanism by which both SeQDs and SeNPs are being processed inside the cells to exert these effects is still undefined.

### 6.3. Se Supplementation in Other Cardiovascular Diseases

Aside from MI and I/R injury, Se supplementation for the prevention of other cardiovascular diseases, such as peripartum cardiomyopathy and coronary heart disease, has been reported, again with mixed results. Patients with peripartum cardiomyopathy (left ventricular ejection fraction < 45%) and Se deficiency (< 70 mg/L) were randomly assigned to receive oral SeMet (200 μg/day) for 3 months [95]. While symptoms of heart failure were reduced in the patients given SeMet, the percentage of left ventricular ejection fraction (LVEF) remained similar between the control group and the Se-supplemented group. It is unknown whether a longer course of SeMet treatment would eventually improve the LVEF; however, the results suggest that heart failure was somewhat mitigated in the Se-supplemented group. Similarly, there have been multiple randomized controlled trials using different forms of Se to mitigate coronary heart disease with mixed results [99]. Meta-analysis of these studies revealed that Se supplementation decreased serum C-reactive protein levels and elevated GPX1, but it had no effect on mortality from coronary heart disease, nor did it alter any lipid profiles. 

## 7. Discussion

Since the discovery of Se involvement in Keshan’s Disease more than fifty years ago [2,23,93], the knowledge of the involvement of Se in cardiovascular diseases has expanded significantly. Se is now known to participate in a host of different cardiovascular disorders, including myocardial infarction, heart failure, cardiomyopathies, atherosclerosis, and coronary heart disease, as Se deficiency is associated with increased risk of these cardiovascular diseases [4,5,30,99,117]. Therefore, it is unsurprising that Se supplementation has been explored as a potential therapeutic to treat several of these cardiovascular disorders when they are linked to a deficient intake status for Se. However, assessing the benefits of Se supplementation has proven challenging as the duration of Se treatment, dose, and the varying pharmokinetics of different chemical forms of Se have made it difficult to assess the therapeutic potential of Se supplementation, at least in clinical studies. Combination studies have also played a role as many clinical studies have combined Se with another nutritional supplement, making it problematic to determine the effects of Se alone. Experimental animal models using Se have been more successful, likely due to the ability to control more factors, such as the environment and diet, among other factors. Due to these complicating factors, it is still unknown whether Se supplementation can be useful as a nutritional supplement for patients with cardiovascular disease. More rigorous studies that focus solely on Se are needed to assess the true therapeutic potential of Se to mitigate cardiovascular diseases, or new targets such as selenoproteins themselves.

Like Se, significant progress has been made elucidating the roles of different selenoproteins in the heart. Selenoproteins serve as antioxidants, regulators of oxidative stress, controllers of calcium flux, and mediators of thyroid hormones. They may also play a role in immune cell migration, contributing to atherogenesis [83]. However, many selenoproteins are still understudied in the heart, particularly in disease situations, and it remains unknown what these proteins are doing in these contexts. There is some evidence that selenoproteins regulate/are regulated by miRNAs [38,72]—post-transcriptional regulators of gene expression that are involved in numerous signaling pathways. The miRNAs and their selenoprotein targets potentially provide hundreds of novel targets for study and it is essential that we learn more about the mechanisms behind selenoprotein and miRNA involvement in cardiovascular diseases, as they could eventually open new avenues for therapies. 

## 8. Conclusions

Much has been learned about the role of selenium and selenoproteins in the heart; however, there remains substantial work to be done, particularly in studying how selenium deficiency impacts selenoproteins during disease conditions. These studies could significantly improve our understanding of how selenium and selenoproteins work on a molecular basis during the pathogenesis of cardiovascular diseases, and potentially lay the foundation for the development of novel therapeutics or improved nutritional guidance.

## Data Availability

Not applicable.
